# Periosteal osteosarcoma of the clavicle: A case report and review of the literature

**DOI:** 10.1016/j.ijscr.2022.107430

**Published:** 2022-07-20

**Authors:** Son Do Le Hoang, Huy Hoang Quoc, Bach Nguyen, Binh Le Nguyen, Duong Tran Binh, Vinh Pham Quang

**Affiliations:** a Department of Orthopaedic, Cho Ray Hospital, 201 Nguyen Chi Thanh Street, District 5, Ho Chi Minh City, Viet Nam; b Department of Orthopaedics and Rehabilitation, University of Medicine and Pharmacy at Ho Chi Minh City, 217 Hong Bang Street, District 5, Ho Chi Minh City, Viet Nam; c Department of Orthopaedics, University Medical Center Ho Chi Minh City, 201 Nguyen Chi Thanh Street, District 5, Ho Chi Minh City, Viet Nam

**Keywords:** Periosteal sarcoma, Clavicle, Claviculectomy

## Abstract

• Periosteal osteosarcoma arising from clavicle is extremely rare with only two cases documented in English literature.
• Diagnosis based on the relation of clinical symptoms, radiology and histology.
• Current treatment included neo-adjuvant chemotherapy and wide resection of the tumor.
• Total claviculectomy without reconstruction can achieved good oncological and functional outcome.

## Abstract (vi)

**Introduction:**

Osteosarcomas arising from the bone cortical surface can be subdivided into parosteal, periosteal, and high-grade surface osteosarcoma. Periosteal osteosarcoma is a rare primary malignant bone tumor arising from the surface of long bones, especially femur and tibia, comprising 4 % of all osteosarcomas. It is an intermediate-grade tumor, and current treatment includes adjuvant chemotherapy and wide tumor resection with limb-salvage surgery. Clavicle is extremely unusual site of periosteal osteosarcoma with only two cases documented in English literature. We report the third case of periosteal osteosarcoma of the clavicle in English literature. In case of primary malignance tumor rising from clavicle, total claviculectomy without reconstruction can be a good treatment option in term of oncology and functional regardless of some shoulder disabilities.

**Case reports:**

A 20-year-old female presented with a less pain mass over her right clavicle. Diagnosis of clavicular periosteal osteosarcoma was made based on clinical features, radiology and histology. Treatment included neoadjuvant chemotherapy by Doxorubicin-Cisplatin followed by tumor wide resection and total claviculectomy. Patient received 3 episodes of chemotherapy with the same agent postoperative. Postoperative course was uneventful. Followed up at 32nd month, the wound heal completely. There is no sign of local recurrence or metastasis. Patient can back to previous works and daily activities with little or no restriction, Constant score of right shoulder is 81.

**Discussion and conclusion:**

Periosteal osteosarcoma (PEO) is a surface osteosarcoma among with parosteal osteosarcoma and high-grade surface osteosarcoma. Periosteal osteosarcoma affect mostly diaphyseal of long bone, especially femur and tibia. Clavicle is the extremely rare position of periosteal osteosarcoma with only two cases documented in English literature. The radiologic characteristic of periosteal osteosarcoma is a soft-tissue mass that causes extrinsic erosion of thickened underlying bone cortex and periosteal reaction extending into the soft-tissue surrounding. Current treatment included neoadjuvant followed by wide tumor resection. Partial, sub-total or total clavilectomy is the treatment of choice in case of periosteal arising from clavicle. Total claviclectomy without reconstruction can achieved good oncological and functional outcome. Surgical treatment prognosis is good with good with 5 year survival rate >80 %.

## 1 Introduction and importance

Osteosarcoma is the most common primary malignant accounting nearly a-third of all bone primary malignant tumor. Periosteal osteosarcoma is a rare variant of osteosarcoma arising from cortical of long bone, mostly from femur and tibia. It is an intermediate-grade tumor and current treatment included neo-adjuvant chemotherapy and wide tumor resection even though the role of chemotherapy still unclear. Wide tumor resection with adequate surgical margin is the cornerstone of treatment of periosteal osteosarcoma. Surgical treatment prognosis is good with survival rate 83 % [1] Clavicle is an extremely uncommon site of periosteal osteosarcoma due to it is the first bone to ossify in human body. The two first documented cases in English literature were reported by Oda [2] and of C. Lim [3]. Our case is the third documented case of clavicle periosteal osteosarcoma treated with total claviculectomy without reconstruction surgery. No local recurrence or metastasis found at 32nd month followed up. Good shoulder function was achieved. Patient can be returned to normal works with little or none restriction.

## 2 Case presentation

A 20-year-old female presented with a less pain mass over her right clavicle, which had been gradually increasing in size over the last 3 months. There was no fever or weight loss and decreased appetite. She has no history of malignancy or trauma, and she has no familial history of tumor also. Local examination revealed a 4 × 6 cm × cm firm, tender mass anteriorly and superiorly over the right clavicle. Tumor was smooth and well defined margin. The tumor is not fixed to the skin but inseparable from underlying clavicle bone. There was no local increase of temperature and erythema. The patient had full shoulder active range of motion (AROM), normal strength compare with unaffected side, and no neurovascular deficit.

**Fig. 1:**
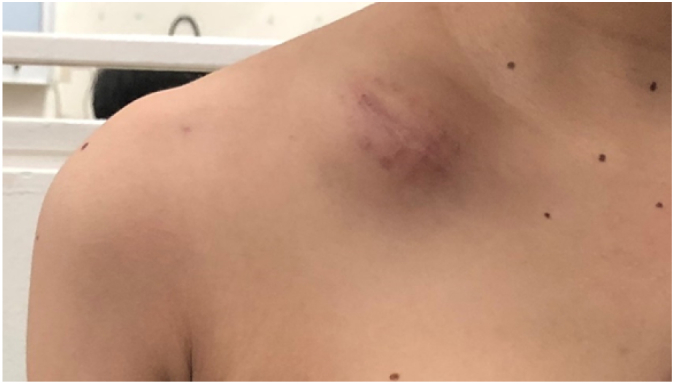
A firm, tender mass anteriorly and superiorly over the right clavicle.

Radiograph demonstrated a defined, surface based lesion arising from the mid shaft of the right clavicle. There was cortical thickening inferior and superior surface of clavicle on AP view. Codman's triangle lesion, irregular and ill-defined lytic bony lesion of mid shaft clavicle outer surface (Fig. 1).

**Fig. 2:**
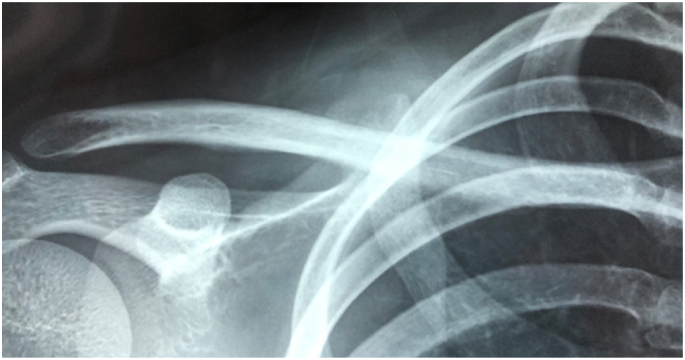
X ray showed a lesion arising from mid shaft of clavicle with periosteal reaction and cortical thickening.

MRI revealed a circumferential soft tissue mass around clavicle, which was low signal intensity on T1W, hyper-intense on T2-W sequences and heterogenous enhanced with contrast. There was a few intramedullary hypointense lesion on T1, hyperintense on T2W and enhanced by contrast agent, suggesting either bone marrow edema or intramedullary invasion of tumor. Chest and abdominal CT-scan does not showed any sign of metastasis (Fig. 2).

**Fig. 3:**
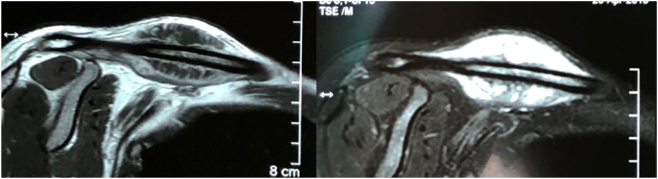
MRI showed hypo-intense signal lesion on T1W, hyper-intense on T2-W sequences and heterogenous enhanced with contrast. Noted that intramedullary hyper-intense on T2.

Open biopsy was performed. Histology showed tumor tissue with hyperplagia of osteoblast with various degrees of nuclear atypia. There was large amount of well differentiation chondro cytes with various sizes. The matrix was composed of mature bone and chondro tissue. These features were considered most consistent with periosteal osteosarcoma (Fig. 3).

**Fig. 4:**
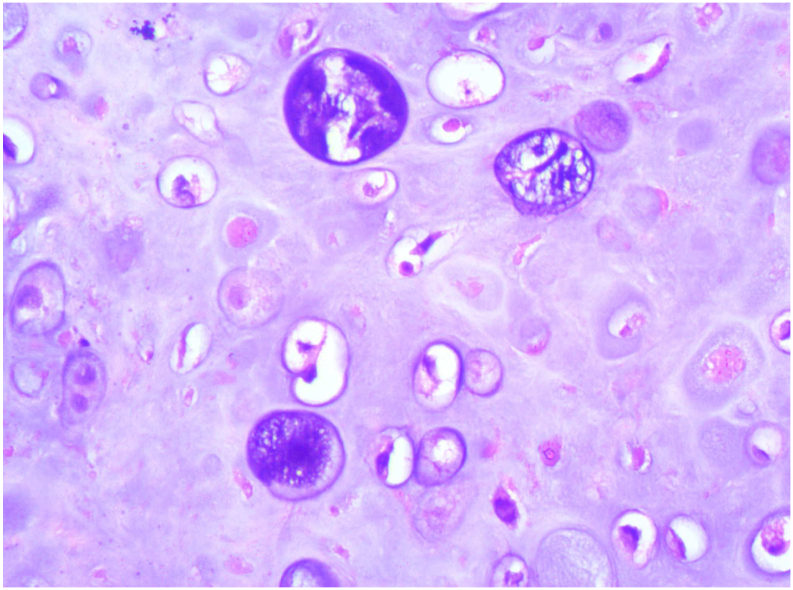
Microscopic image showed large amount of well differentiation chondrocytes and osteoblast hyperplagia.

Diagnosis was made based on clinical symptoms, imaging and histology. The patient underwent three episodes of preoperative chemotherapy by Doxorubicin-Cisplatin. There was no remarkable change in size of the lesion after neo-adjuvant chemotherapy. A right total clavicle resection was performed by the first author – an experienced surgeon in orthopaedic oncology. No-touch technique was applied to avoid violation of the tumor mass. Local wide resection of tumor and surrounding tissue with respect to sub-clavicle neurovascular structure. The tumor was split along its long axis showing a 31 × 34 × 73 mm whitish tissue surrounding the mid shaft clavicle cortex with few focal necrosis. Post-operative histology reconfirmed diagnosis of periosteal osteosarcoma with significant tumor necrosis resulting from neo-adjuvant chemotherapy. The patient's right shoulder was immobilized post-operatively and pain-killer was given to her. The post-operation was uneventful. The wound healed with no complication. A further 3 episodes post-operative chemotherapy using the same agents was completed (Fig. 4).

**Fig. 5:**
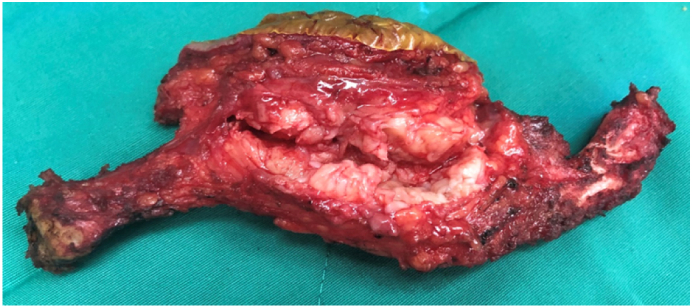
Total right claviculectomy with wide resection of tumor.

**Fig. 6:**
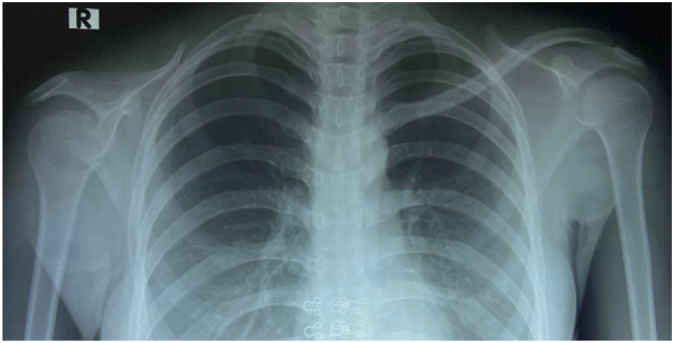
X ray showed R scapular and humeral bone lower than the left one. No sign of local recurrence found.

The patient had been reassessed at 1-2-3 6-12-24 and 32 month post-operative. At 32nd month, no evidence of local or metastasis was found on clinical examination and CT-scan (chest and abdominal). Pain free, full active range of motion was achieved. Patient could returned to work with no limitation. Patient usually felt uncomfortable when grasping heavy object for several minutes or lifting object weight >3 kg. Clinical observation and X ray revealed Right scapula and humerus head dropped in relatively with Left shoulder. The Constant Score was 81 and 90 in affected and unaffected side respectively (Fig. 5, Fig. 6).

## 3 Method

This case report is compliant with the SCARE Guideline 2020 [4].

## 4 Clinical discussion and conclusion

### 4.1 Overview of periosteal sarcoma

Periosteal osteosarcoma (PEO) is a surface osteosarcoma among with parosteal osteosarcoma and high-grade surface osteosarcoma. Ewing was the first one to describe this disease in 1939 and Lichenstein reported it as an opposite one of inter-medullary osteosarcoma [5]. Periosteal osteosarcoma is also called as juxtacortical chondrosarcoma by some authors and is accounted for about 25 % of surface osteosarcoma cases and only 1.5–2 % of all osteosarcoma [1]. However, WHO classification does not recommend to call it as juxtacortical chondroblastic osteosarcoma [6]. It affect mostly on the diaphyseal and meta-diaphyseal region of long bone, especially tibia, femur and humerus, and sometime mandible. PEO incidence is reported to be high in male gender and in the age group between 20 and 40 years old [7]. The PEO of clavicle our study is the third case which is reported on English literature among 2 case report article of Oda [2] and C. Lim [3]. Clavicle is the rare position of periosteal osteosarcoma because the clavicle is the first bone to ossify in human body. It occurred through intermembranous ossification about fifth to six week of fetus life by two primary center, lateral and medial, and after that in one secondary center, stenum in 18–20 year-old human. These centers unite at about 25 years of life [8].

### 4.2 Diagnosis

The clinical presentation of periosteal sarcoma is usually pain and swelling in the site of tumor. The tibial meta-diaphysis, especially proximal third is the most affected region, following the middle and distal third of femur [7]. In order to diagnose periosteal osteosarcoma and differentiate it with other type of tumor, such as parosteal osteosarcoma and high-grade surface osteosarcoma, radiologic and histologic measures have to be obtained. On radiograph of Xray and computated tomography (CT), periosteal osteosarcoma is decrbibed as a cortically base tumor with a broad-based soft tissue mass. The underlying thicken cortex is usually seen erosion or scalloping making a shallow crater called as saucerization. The periosteal reaction is often perpendicular and spiculated to the cortex known as sunburst appearance, beside other sign such as Codman's triangle. The extent of minerization in the soft tissue is about 50 % of cortical circumference with many degrees (mild, moderate, marked). The medullary involvement of PEO is also reported. On MRI, hypointense of tumor is found on T1w. On the other hand, on T2w images, the tumor has hyperintense signal and presents a septonodular pattern of enhancement because of chonroblastic content [9]. Histopathology characteristic of PEO can be seen as various sized lobulated islands of atypical hyaline cartilage and areas of moderately high-grade spindle cells located peripherally [10].

### 4.3 Treatment options

Because of the diaphyseal or metadiaphyseal location of the tumor and the advance of surgical technique, wide resection is the method of choice in PEO management. Amputation is only preserved for big and medullary invaded tumor or in case of recurrent after primary resection [7]. For clavicle maglinancy tumor, the treatment of choice is clavicle resection or claviculectomy which can be partial, subtotal or total claviculectomy. The extent of resection depends on whether medullary is involved, and subtotal claviculectomy is thought to be ideal for shoulder circle stability [11], [12]. Reconstruction of clavicle after claviculectomy is also debatable and some authors support the claviculectomy alone method with the same functional outcome and with shorter operating time and less postoperative comordities. A systematic review done by X. Yu et al. reported 11 studies with 70 cases of both total and subtotal claviculectomy in which 14 cases went under clavicle reconstruction and 56 cases under isolated claviculectomy. After a mean follow-up duration of 53 months, the limb function and the complication rate were similar when comparing 2 groups. The isolated claviculectomy had fewer procedure and faster recovery [13].

The role of chemotherapy is inconclusive, both with neo-adjuvant and adjuvant therapy. Revell et al. reported 17 cases with periosteal osteosarcoma under tumor resection and patients went under chemotherapy if tumor showed high grade histological features or if medullary involvement was present. Among these patients, 14 patients were received chemotherapy with dororubixin and cisplatin and 3 patient did not enrolled in the therapy. In the chemotreated-group, 10 patients also received neo-adjuvant chemotherapy while 4 patients only received adjuvant chemotherapy. After the mean follow up of 52 months, none of 17 patients had distal metastasis, and two patient had good respond to neo-adjuvant chemotherapy (90 % necrosis), tumor-related death was not reported. The authors of this study recommended the use of neo-adjuvant chemotherapy for high-graded tumor and medullary involvement [14]. Cesari et al. reported that 33 patients with periosteal osteosarcoma, and among these 14 patients received chemotherapy for grade 3 tumors (4 of them received neo-adjuvant chemotherapy). Criteria for chemotherapy were not reported, and protocol of chemotherapy was not fix among patients. There was no difference in the ten-year overall survival rate of those who received chemotherapy versus chemotherapy-free patients (86 % vs. 83 %; *P* = 0.73) [15]. In our case, because of medullary involvement, patient was under neoadjuvant therapy with doxorubicin and cisplatin, and recorded necrosis of tumor after claviculectomy. After surgery, adjuvant chemotherapy was applied for 3 months and no sign of recurrent or distal metastasis.

### 4.4 Prognosis of surgical treatment

Prognosis of periosteal osteosarcoma depends on many factors such as medullary involvement, the time of diagnosis. In general, PEO metastates mostly to lung and pleura [7]. However, PEO has the low incidence of metastasis resembling parosteal osteosarcoma. In a case series of 119 patients with PEO, Grimer et al. noted metastasis in 17 patients (14 %) [15]. Wide resection with adequate can be enough for tumor control. Rose found that all patients with marginal or intralesional margins had local recurrences. Surgical treatment prognosis is good with survival rate >80 % in 15 years followed up [1].

### 4.5 Clinical function after total clavicle excision

Wide local resection is recommended treatment for periosteal osteosarcoma of the long bone. The current treatment is a combination of adjuvant chemotherapy and limb-preserve surgery. In case of tumor arise from clavicle, total claviculectomy procedure is a good option if a definitive treatment is the target for clavicle malignant management. Claviculevtomy is relative rare procedure, it can be used in some condition like infection, trauma or tumor… Clavicle bone provides structural, protective and cosmetic function. Traditionally, clavicle, fibula and rib are called expendable bones. Some reports have documented good oncological and functional outcome with subtotal or total claviculectomy and most of authors recommended resection without reconstruction. Bio-mechanic testing in patient after en-block claviculectomy revealed some weakness in shoulder abduction, flexion, and adduction but not in internal rotation, external rotation, or extension. These weaknesses may be clinically insignificant to the patient, depending on the level activities of daily living [17]. Considering previously mentioned protective function of the clavicle, Li et al. found no chronic neurovascular bundle injury caused by loss of the clavicle occurred in his series. They also found that there were no significant differences in Constant-Murley score between reconstruction and non-reconstruction group. Reconstruction after claviculectomy have some advantages in defect reconstruction, neurovascular bundle protection, and cosmetic aspect but can accompany with higher risk of infection, secondary surgery, nonunion or delay rehabilitation post-operative [11]. Rubright found deficits in strength were present in the aclavicular limbs but patients compensate for loss of the clavicle with minimal functional deficit. DASH scores and SF-36 scores were not significantly inferior to normal population while Constant score and UCLA score lower than unaffected side. Despite some deficits, these patients continue to have normal self-perceptions of overall health and global upper extremity function [18]. In our case, total claviculectomy without reconstruction give good shoulder function. Patient does not fell any difficulty when using shoulder in normal works or daily activities. Total claviculectomy, therefore can be used in case of malignances of clavicle with good oncology and functional outcome postperative.

## Sources of funding

The authors received no financial support for the work, authorship, and/or publication of this report.

## Ethical approval

This report was conducted in accordance with the World Medical Association Declaration of Helsinki.

## Consent

Written informed consent was obtained from the patient for publication of this case report and accompanying images. A copy of the written consent is available for review by the Editor-in-Chief of this journal on request.

## Author contribution

Son Hoang Do Le: conceptualising the plan for surgery, performing the surgery, follow up patient's recovery, writing the literature review for case report, reviewing the manuscript.

Huy Hoang Quoc: Assisting in planning and in the surgery, writing the draft for case report.

Bach Nguyen: Assisting the surgery, writing the literature review, taking note of postoperative function.

Binh Le Nguyen: taking note and data visualisation perioperatively.

Vinh Pham Quang: reviewing the paper, Assisting the surgery, prepare the neccessary equipments: Analyzing the radiology and MRI.

## Research registration

None.

## Guarantor

Vinh Pham Quang MD, PhD.

## Provenance and peer review

Not commissioned, externally peer-reviewed.

## Declaration of competing interest

The authors declare no conflicts of interest in this work.
